# Carbolic Acid and Tansy Poisoning

**Published:** 1901-12

**Authors:** Charles E. Boynton

**Affiliations:** Atlanta, Ga.


### CARBOLIC ACID AND TANSY POISONING.*

By CHARLES E. BOYNTON, M.D.,

ATLANTA, GA.

*Mr. President and Gentlemen :*

I have chosen as a subject for a paper to-night two poisons, the one being, I think, the drug most frequently chosen as a means of leaving this earth, and the other being very frequently taken to prevent the coming of an undesired visitor.

The cases of the two poisons are, in a way, rather closely connected, for the one, carbolic acid, is more frequently taken by young, unmarried pregnant women, remorseful for the first offense, than by any other class. The other, tansy, is more frequently taken by older women, the knowing ones, either married or unmarried, and not with the idea of suicide, but simply to get rid of an unwelcome boarder. They do not realize the risk they run.

*Carbolic acid poisoning* is of two types—the one slow, the other rapid.

*The slow form* is usually due to the local use of the drug, although after large, strong dressings the patient may sink rapidly into profound shock, followed by death.

Slow poisoning usually follows the prolonged use of wet carbolic acid dressings of various strengths. The first symptom is usually a smoky appearance of the urine. Hence, in all cases where carbolic dressings are used, the urine should be carefully watched.

*Read before the Atlanta Medical Society, November 7, 1901.

If this warning is not heeded, the patients become languid, lose their appetite, become nauseated and vomit, or they may be troubled by recurring attacks of vomiting. They suffer from more or less headache and dizziness; bronchial irritation, which in some cases become very severe; often there are severe pains in the region of the kidneys, pruritus or various paresthesia, and loss of power in the legs. In addition there is a gradual loss of strength. Some authors speak of a marasmus, or chronic poisoning, due to the surgical use of the drug.

The acid is very readily absorbed, whether taken internally, applied to the unbroken skin, or to wounded surfaces, or even from air impregnated with the drug.

Dr. Falckson, after two hours' exposure to carbolic acid spray, recovered 30 grains of carbolic acid from his urine.

A fatal case has been reported from dressings applied to a wound four inches long. A single vaginal injection has produced very serious constitutional symptoms. The local application has, in a number of cases, been followed by severe local gangrene, and especially is this so when applied to the fingers or toes. This goes to show that the remedy, even in weak solution, if long applied, is not without dangers, and should make the surgeon choose some other dressing, and the laity should not be allowed to buy the drug at all without a doctor's prescription. In nearly all the cases of gangrene it has been used without consulting a physician.

The *treatment*, in cases of this class, is to remove the dressings and thoroughly cleanse the wound. Of course the patient should be kept in bed, and the food should be light but of the most nourishing kind. Alcohol in some form should be given, together with strychnine or other stimulants, in sufficient quantities to overcome any depression and to enable the patient to throw off the poison. The bowels should be kept gently open by a soluble sulphate, as magnesium or sodium sulphate, as these form the harmless sulpho carbolates.

Fluids should be allowed liberally if the stomach will retain them. It is also a good plan to give normal salt solution either by the bowel or under the skin, as the case may require. The acid is rather rapidly thrown off through all the excretories in about 36

to 48 hours, so that, if the strength of the patient can be kept up, the chances of recovery are good.

The *rapid form from local* use is often very puzzling, as it usually occurs after major operations, and hence is very difficult to distinguish from surgical shock. For these cases the wound should always be cleansed and the shock treated.

When taken *internally* carbolic acid is very rapidly absorbed, and, if the dose is sufficient, the symptoms of poisoning very soon appear.

In some cases the effect is so rapid that the patient drops to the ground as if seized with a "stroke of apoplexy." More frequently, however, from 15 to 30 minutes elapse before the symptoms are marked.

If taken in concentrated form, with the idea of suicide, we usually find local evidence in the shape of strong odor of the drug, and whitened areas of skin and mucous membrane around the face and mouth. These whitened areas later become hard, tough, and of a reddish-brown color. This condition is due to the power of the acid to coagulate the albumin of the tissues. These eschars are more or less in evidence according to the way the acid is taken and to the type of the patient. The nervous subject is very apt to spill the acid about the face, therefore the marks are plain; in deliberate cases the only marks are found in the mouth.

I had one patient who had taken the acid through a glass tube, therefore the eschars were confined to the back of the throat and the odor was very faint. This patient, brought to the hospital at night, denied taking the acid, and the local evidence was not found until next day in a good light. However, the marked symptoms soon appearing, the stomach tube was passed and the strong acid was found. This woman was about three months pregnant.

Immediately after taking the acid a burning sensation of short duration is experienced in the mouth, esophagus and stomach. There is some nausea and vomiting at first, but this is soon followed by a complete inability to vomit, even after taking emetics. They complain of dizziness; seem weak and unsteady on their feet; the pupils contract more or less; the pulse becomes rapid and more feeble, but at this time is still regular; respiration is embarrassed. Collapse, with unconsciousness, quickly follows; the skin is cold and clammy; the face is pale, becoming cyanosed as the disturbance of respiration becomes more marked; the pulse becomes still faster and loses in strength continuously, finally becoming irregular, intermittent and almost imperceptible. In some cases, however, the pulse becomes very *slow*, instead of rapid. The breathing, at first, is more rapid, bnt at the same time is more shallow; it becomes less and less deep, taking on a gasping character, and finally is a mere rattle in the throat, stopping altogether while the heart is still beating. In some cases stertorous breathing is present. The mental condition is first dazed, passing into stupor, and finally into utter insensibility. The temperature is constantly lowered. The reflexes, by the ordinary tests, are abolished, but the muscles still respond to the galvanic current. There is often retention of the urine, and it may be suppressed. If drawn with the catheter it may appear normal in color, but usually turns smoky or greenish in color if allowed to stand, provided death is delayed long enough for urine to be formed after the poison is taken. It always shows a great reduction or absence of sulphates.

Female patients are usually pregnant If the case does not prove fatal the acid is rapidly eliminated by all the excretory organs, and the patient is left to recover from the local burns. There may be more or less stenosis of the esophagus, together with gastric disturbances and also pain.

The *diagnostic points* are the odor of carbolic acid about the patient; the white or reddish-brown eschars about the face and mouth; the quick appearance of collapse, with the cold, clammy skin, rapid and feeble pulse, gasping respiration, and total unconsciousness, together with the dark, smoky urine, with absence of sulphates.

The *prognosis* is grave, but not hopeless if the treatment is begun soon and carried out in a vigorous and heroic manner.

The *treatment* must be rapid, as a few minutes may turn the scale for or against the patient.

The clothing should be quickly removed and the patient wrapped in blankets and surrounded with hot-water bottles. Elevate the foot of the bed in order to keep as much blood around the heart as possible.

A stomach tube is quickly passed and the stomach thoroughly washed with a concentrated solution of one of the soluble sulphates which are the chemical antidotes. A good dose of salts should be left in the stomach.

Stimulants should be freely given with the hypodermic needle and strong black coffee given through a long rectal tube.

Normal salt solution should be given either under the skin or intravenously.

Artificial respiration is begun whenever indicated.

Oils should not be used as they dissolve the acid.

The after treatment should be directed to keeping the mouth clean, giving some drenchant, as syrup of ocacia and morphine, to quiet any pain.

The diet should be liquid and nourishing.

Later esophageal bougies should be passed if any evidence of stenosis appears.

Recently the washing of the stomach with alcohol has been brought forward as a remedy, but with this I have had no experience.

CASE :——, female, 22 years old and weighing 150 pounds, was brought to the hospital in the ambulance about 6 o'clock in the evening. The patient had had a quarrel with her lover and had taken two ounces of pure carbolic acid with the idea of ending her life.

She had evidently acted with deliberation, as, externally, the only evidence of carbolic acid was on the lips and a small burned streak running down the chin.

The ambulance surgeon had given strychnine, grain one-thirtieth hypodermically, on the way in.

I saw the patient about two minutes after she was placed in the Prison Ward and found her in an extremely grave condition.

The skin was cyanosed, cold and clammy. The lips, fauces, tongue, pharynx, in fact, the whole of the mouth and throat were whitened and excessively congested and swollen, while the esophageal opening was closed.

The radical pulse was not to be felt at all, but a very weak, irregular and intermitting pulse could be felt in the brachial.

The heart's action, as determined by the stethoscope, was weak, irregular and intermitting, while the heart sounds were feeble. In fact, the heart was making that last effort so often seen before death.

The breathing was shallow, slow, gasping, only about five or six per minute and accompanied by a marked tracheal gurgle or rattle.

The breathing was practically no breathing at all.

The patient was absolutely unconscious, limp and lax.

The temperature I did not have time to take, so don't know what it was.

The pupils were dilated.

The urine, obtained later, was dark and smoky.

I think five minutes longer without treatment would have ended life.

Gentlemen, this is no overdrawn picture, but simply as it was in this case.

*Treatment.*—Fortunately I had plenty of assistance at hand and everything in readiness for treating poison cases, so things were done in much less time than it takes to tell it.

Artificial respiration was begun immediately.

As soon as possible the patient was given strychnine, grain one-twentieth and nitroglycerine, grain one-twenty-fifth hypodermically.

The foot of the bed was raised about two feet.

Strong bandages were tightly drawn, beginning at the toes and carried up to the body, thus cutting off the blood from the lower extremities as much as possible, and, together with the elevation of the foot of the bed, keeping the blood around the heart.

At the same time my senior assistant, Dr. Joy, was trying to pass the stomach tube, but said the swelling was so great that the tube would not pass.

I inserted my finger into the pharynx and found the opening into the esophagus closed.

I then guided the tube over the larynx and poured about one drachm of saturated Mg $SO_4$ solution into the funnel and waited; about a moment later the tube slipped down one-half to one inch. More solution was poured in and the tube slipped a little farther each time—finally reaching the stomach.

The contents of the stomach were then siphoned off and the globules of carbolic acid could be seen returning at first. This washing was continued until the return solution was perfectly sweet, about five gallons of water and two pounds of epsom salts being used in the operation.

One ounce of salts was left in the stomach.

Hot water bags to the number of eight or ten were placed around and the patient covered with blankets.

One pint of hot, strong, black coffee was given through a long rectal tube.

All this time stimulants were given freely by hypodermic injection.

A syringe full of whiskey was given every three minutes at first and then at longer intervals.

About ten minutes after the first dose of strychnine and nitroglycerine I gave strychnine, grain one-twentieth; atropine, grain one-fiftieth; nitroglycerine, grain one-fiftieth; then various stimulants at intervals as the pulse and breathing were improving.

The stimulation was continued for forty-five minutes, when slight jerking movements in the arms were present; in other words, the full physiological action of strychnine had been reached. She was then given morphine, grain one-fourth.

Two hours after applying the bandages, they were gradually loosened, allowing the blood to return to the lower limbs.

About four hours after the poison had been taken, the patient was semi-conscious and the next morning her mind was perfectly clear. Her throat and mouth were very sore. She could swallow very slowly. She was given syrup of acacia to swallow and also to hold in her mouth almost constantly. The salts caused free movement of the bowels toward morning.

The further course was uneventful and she was able to leave the hospital in about two weeks.

In all strychnine, one-fourth gr.; nitroglycerine, one-fifth gr.; atropine, one-twenty-fifth gr.; ether one-and-one-half drams; spirits camphor, twenty minims; digitalin, one-fiftieth gr.; whiskey, one-and-one-half ounces, and morphine one-fourth grain were given by hypodermic needle.

Gentlemen, I report this case for several reasons:

1. It was one of the most interesting and gratifying cases I have ever treated.

2. It shows that while there is life there is hope.

3. It shows that under certain conditions patients will stand and are benefited by heroic stimulation.

4. This case could never have been saved in private practice.

5. The text books advise against the use of the stomach tube in cases of corroding poisons in general, and some say it is contra-indicated in carbolic acid poisoning; their reason being that it may cause a perforation.

In the first place, often in desperate cases, emetics will not work and even when they do, the work is not thorough.

I always use a soft rubber stomach tube in every case of poisoning, and have yet to see the slightest harm resulting from this practice, while I have seen a number of cases come through who otherwise would not have done so.

If the stomach is so badly eroded that a soft tube properly passed will perforate the stomach wall, the patient is beyond hope anyway, so I say give him his best chance and that is the stomach tube.

---

My first intention was to give a general review of tansy poisoning, but finding almost no literature on the subject, I have decided simply to report the following case:

CASE OF TANSY POISONING.

Mrs. ———— ———, 45 years old, weight about 150 pounds, and the mother of six children. On February 19, 1900, about 4:30 p.m., took about three drachms of the oil of tansy, as I found out afterwards. About 5:15 p.m. she was seized with a convulsion.

I first saw her about 6 p.m. She was sitting up in bed and seemed to be in a semi-dazed condition, answering questions with difficulty, as if her mind did not grasp things readily.

She said that about 4 p.m. she had a little pain in the stomach, and had taken a little peppermint water (proved to have been oil of tansy). After this pain in the stomach had increased, she felt dizzy and did not remember anything further until she awoke and found herself in bed. Those in the room told her she had had a "fit" and upon questioning they described an epileptic form convulsion. They said she began to foam at the mouth, stiffened out and then began to jerk all over the body, and after this she relaxed and lay unconscious for about ten minutes before coming to. I examined her about this time.

Patient seemed dazed and stupid; pulse 100, of good size and strength; respiration 24; temperature not taken. Otherwise physical examination showed heart and lungs to be normal. Epigastrium a little sensitive to pressure. Patient began complaining of nausea and in a few moments vomited a little partially digested food smelling strongly of tansy. Very soon after this patient was seized with another convulsion. She threw out her arms as if to call her husband, who was standing near, and almost immediately fell backwards on the bed. Eyes rolled from side to side; pupils dilated. She became perfectly rigid, with tongue protruding and caught firmly between the teeth. Frothing at the mouth. During this period of rigidity the chest was fixed in deep inspiration; pulse of fair strength and about 120 per minute; cyanosis became marked.

After about 20 to 30 seconds this tonic spasm relaxed and was followed by a number of clonic spasms in quick succession, during which there were violent jerking movements of the lower jaw and all the extremities. These clonic convulsions ceased after two or three minutes and left the patient in a relaxed and collapsed condition, with stertorous breathing and unconsciousness.

In something like four or five minutes a third convulsion, like the one described, came on and was followed by a fourth very soon afterwards. At this time patient was in a condition of extreme shock and absolutely unconscious. Pulse very small, feeble, 150 per minute and almost imperceptible. Respiration between 30

and 35, very shallow and accompanied by marked stertor. Heart-beat rapid, feeble, sounds faint. Extremities cold as far as elbow and knee. Forehead clammy. Temperature not taken.

About 8:30 p.m. patient had rallied and seemed to recognize people in the room, but was very much dazed.

Her pulse had dropped to 135 and had gained in strength and volume but was irregular and intermittent. Respiration 25. Extremities becoming warm. Temperature 98 4-5 axilla. Complained of intense headache.

By midnight pulse had fallen to 90 and was of good strength and regular. She was lying in a stupor most of the time, and did not become fully conscious of her surroundings until the next afternoon (February 20, 1900), and did not remember anything since just before the first convulsion the day before.

*Treatment.*—Stomach thoroughly washed by means of soft rubber stomach tube and funnel, about 4 gallons of bicarbonate of soda solution being used; the last gallon returning clear and free from odor of tansy. Hypodermics of strychnine, atropine and morphine. Tongue protected as much as possible. Hot-water bottles to body. Surface rubbing. Blood cut off from lower extremities as much as possible by tight bandaging. Two half-ounce doses of epsom salts one hour apart.

NOTE—February 20.—Patient complains very much of intense headache, which is now confined to the temporal region on both sides, and also of pain in the abdomen. Says arms and legs, and in fact, whole body, feel bruised and sore. Tongue is swollen to about twice normal size and is very sore. Superficial ulcers present.

NOTE—February 21.—Tongue only slightly swollen; coated white; ulcers about healed. Headache much less severe. Pain and tenderness in abdomen, but this passed off during the day. There is soreness of nearly every muscle in the body, especially in arms and legs.

Gradually general improvement took place, and in a few days the patient was herself again.

*After Treatment.*—Hot cloths to the abdomen. Ice-bags to head. "Tongue bath" of syrup acacia, and also to be swallowed. Frequent cleansing of mouth with boric acid solution. Bowels kept gently open; milk diet; massage of muscles.

NOTE—March 8.—Called at 5 P.M. This morning patient aborted fetus about 3½ months old. Placenta and everything said to have come away. Temperature 100 1-5.

Two days later patient showing signs of mild sepsis. I curetted and removed a small necrotic placenta. Made *perfect* recovery.

